# HULC long noncoding RNA silencing suppresses angiogenesis by regulating ESM-1 via the PI3K/Akt/mTOR signaling pathway in human gliomas

**DOI:** 10.18632/oncotarget.7418

**Published:** 2016-02-15

**Authors:** Yu Zhu, Xuebin Zhang, Lisha Qi, Ying Cai, Ping Yang, Geng Xuan, Yuan Jiang

**Affiliations:** ^1^ Department of Clinical Laboratory, Tianjin Huanhu Hospital, Tianjin Key Laboratory of Cerebral Vessels and Neural Degeneration, Tianjin, China; ^2^ Department of Pathology, Tianjin Huanhu Hospital, Tianjin Key Laboratory of Cerebral Vessels and Neural Degeneration, Tianjin, China; ^3^ Department of Pathology, Tianjin Medical University Cancer Institute and Hospital, The Key Laboratory of Tianjin Cancer Prevention and Treatment, Tianjin, China; ^4^ Tianjin Neurosurgery Institute, Tianjin Huanhu Hospital, Tianjin Key Laboratory of Cerebral Vessels and Neural Degeneration, Tianjin, China; ^5^ College of Clinical Laboratory, Tianjin Medical University, Tianjin, China; ^6^ Department of Immunology, Tianjin Key Laboratory of Cellular and Molecular Immunology, Key Laboratory of Educational Ministry of China, School of Basic Medical Sciences, Tianjin Medical University, Tianjin, China

**Keywords:** lncRNA, HULC, glioma, anoikis, proliferation

## Abstract

Tumor angiogenesis plays a critical role in the tumor progression. Highly upregulated in liver cancer (HULC) is a long noncoding RNA (lncRNA) that acts as an oncogene in gliomas. We found that HULC, vascular endothelial growth factor (VEGF), and ESM-1 (endothelial cell specific molecule 1) expression and microvessel density were positively correlated with grade dependency in glioma patient tissues, and that HULC silencing suppressed angiogenesis by inhibiting glioma cells proliferation and invasion. This process induced anoikis and blocked the cell cycle at G1/S phase via the PI3K/Akt/mTOR signaling pathway, thus regulating the tumor-related genes involved in the above biological behavior in human glioma U87MG and U251 cells. However, these effects were reversed by ESM-1 overexpression, suggesting a mediating role of ESM-1 in the pro-angiogenesis effect of HULC. Our results define the mechanism of the pro-angiogenesis activity of HULC, which shows potential for application as a therapeutic target in glioma.

## INTRODUCTION

The highly up-regulated in liver cancer (HULC) human long noncoding RNA is multifunctional and is implicated in various cellular processes, including cancer [1]. Clinically, up-regulation of HULC has been detected in many human malignancies, such as esophageal cancer, osteosarcoma, pancreatic cancer and hepatocellular carcinoma [2–5].

Tumor cells are plastic and can alter cell markers and functions to adapt to the microenvironment, including generating vascularization through vasculogenic mimicry (angiogenesis). Angiogenesis is a common feature of all cancers and is associated with tumor grade and malignancy. Angiogenesis is also accompanied by the inhibition of tumor apoptosis and the induction of invasion by pro-angiogenesis factors. For example, vascular endothelial growth factor-A (VEGF-A) is a pro-angiogenesis gene that is associated with tumor angiogenesis and tumor malignancy, and it also induces tumor proliferation, migration invasion and survival.

Anoikis resistance is a key factor in tumor aggravation and angiogenesis and it was coined by Frisch et al. in 1994. The term “anoikis” comes from the Greek word for “homelessness” and refers to the cell apoptosis response to the absence of a cell-matrix interaction, acting as a critical mechanism in preventing tumor invasion. Accordingly, tumor cells colonizing distant organs escape anoikis [6–10].

However, the role and mechanism of HULC in anoikis, invasion and angiogenesis in gliomas are not fully understood. We found that the levels of HULC, VEGF, ESM-1 (endothelial cell specific molecule 1) and microvessel density (MVD) (CD34) were correlated in glioma patient tissues of various grades. Therefore, we hypothesized that HULC plays an important role in angiogenesis as well as tumor aggravation *in vitro* and *in vivo*. To verify this hypothesis, we established HULC-silenced U87MG and U251 cell lines, and the results showed that (1) HULC silencing suppresses ESM-1-mediated proliferation, adhesion, migration, invasion and angiogenesis through the cell cycle and anoikis regulation in the glioma U87MG and U251 cell lines and (2) HULC is an oncogene in the PI3K/Akt/mTOR signaling pathway and up-regulates the expression of ESM-1.

## RESULTS

### HULC correlates with ESM-1 levels, angiogenesis and tumor grade in glioma patients and cell lines

**Figure 1:**
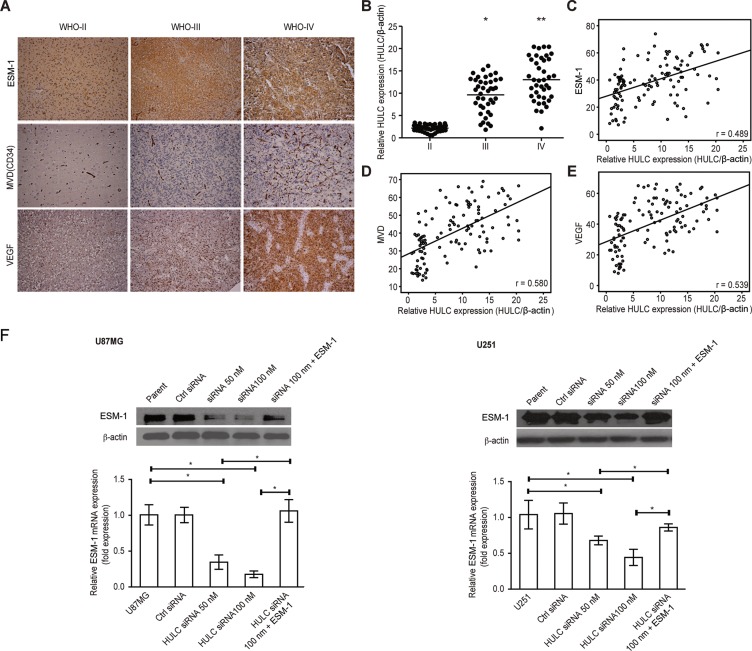
The correlation of HULC, ESM-1, VEGF, and microvessel density in pathological tissue from glioma patients (**A**) The expression of ESM-1, VEGF and microvessels (CD34) in pathological tissue from glioma patients with various WHO grades (× 200). (**B**) The expression of HULC in pathological tissue from glioma patients with various WHO grades. *compared with grade II, *P* < 0.05; **compared with grade III, *P* < 0.05. (**C**) The correlation between the expression of HULC and ESM-1 in pathological tissue from glioma patients. (**D**) The correlation between the expression of HULC and ESM-1 in pathological tissue from glioma patients. (**E**) The correlation between the expression of HULC and ESM-1 in pathological tissue from glioma patients. (**F**) The effects of HULC silencing on the protein and mRNA expression of ESM-1 in the glioma U87MG and U251 cell lines. Error bars, ± SD; *P < 0.05.

We studied the levels of HULC, ESM-1 and angiogenesis (CD34 and VEGF) in the tissues of glioma patients. There were low levels of HULC, ESM-1, VEGF and MVD (CD34) in early-stage tumors (WHO grade II); however, there were higher levels of the above genes in tumors of a more malignant stage (WHO grade III-IV), as shown in Figure 1A and 1B. The levels of ESM-1, MVD (CD34) and VEGF associated with various glioma grades are listed in Table 1.

**Table 1:** The levels of ESM-1, MVD (CD34) and VEGF associated with various glioma grades

	Case		Label index (%)		
Gene	Groups	*n*	Min	Max	Mean
HULC /β-actin	II	40	0.6	3.2	2.2 ± 0.75
	III	40	1.8	16.1	9.6 ± 3.9^*^
	IV	40	5.9	20.4	12.0 ± 4.8^*, **^
MVD (CD34)	II	40	13.6	46.2	28.0 ± 9.4
	III	40	19.2	58.1	40.1 ± 10.5^*^
	IV	40	33.0	66.5	52.6 ± 10.3^*, **^
ESM-1	II	40	7.0	55.0	30.2 ± 12.1
	III	40	10.0	65.0	39 ± 15.7^*^
	IV	40	23.0	74.0	47.1 ± 13.1^*, **^
VEGF	II	40	8.0	46.0	26.9 ± 11.9
	III	40	19.0	62.0	41.4 ± 11.5^*^
	IV	40	35.0	66.0	52.4 ± 9.5^*, **^

* compared with grade II, *P* < 0.05;

** compared with grade III, *P* < 0.05.

After calculating the correlations among the expression levels of HULC, VEGF, ESM-1 and MVD (CD34), we observed a significant linear correlation between HULC, VEGF, ESM-1 and MVD (CD34) in primary glioma patient pathological tissue (Figure 1C–1E). Therefore, we hypothesized that there is a positive correlation between HULC, ESM-1 and angiogenesis.

Next, we explored whether ESM-1 expression was affected by HULC and we found that the expression of ESM-1 was suppressed by HULC silencing, however, ESM-1 expression was significantly up-regulated by ESM-1 expression plasmid was transfected in the U87MG and U251 cell lines (Figure 1F).

### HULC silencing suppresses the potential for the proliferation and angiogenesis of glioma cells *in vitro*

**Figure 2:**
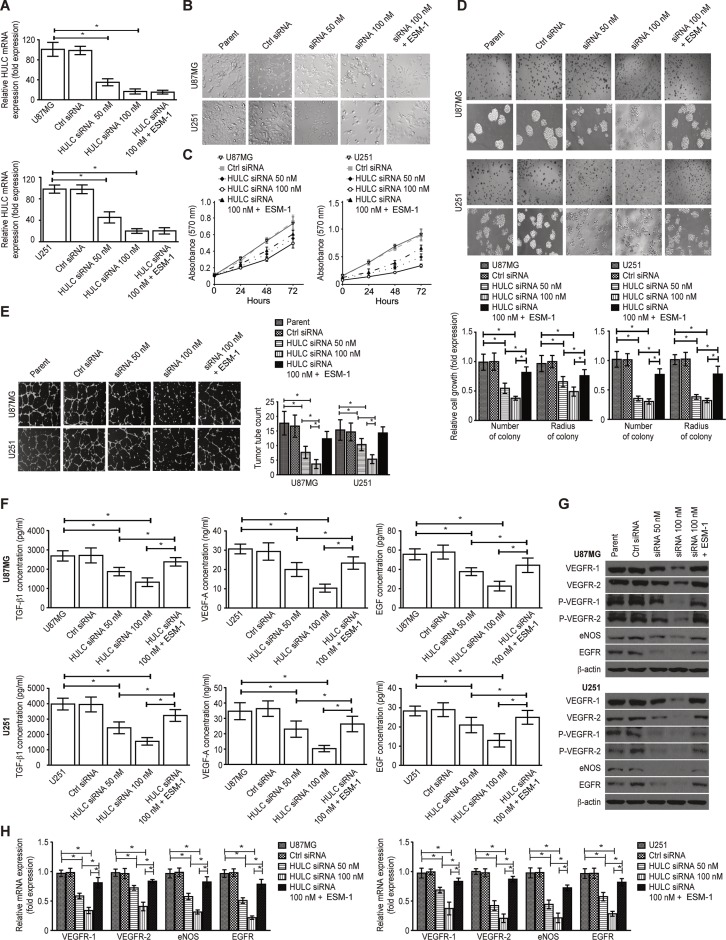
Effect of HULC on the potential of proliferation and angiogenesis of glioma cells *in vitro* (**A**) The effects of the HULC siRNA plasmid and the ESM-1 expression plasmid on the expression of HULC in glioma U87MG and U251 cell lines. (**B**) The effect of HULC silencing on the cellular morphology of the glioma U87MG and U251 cell lines. (× 400) (**C**) The effect of HULC silencing on the proliferation potential of the glioma U87MG and U251 cell lines. (**D**) The effect of HULC silencing on the anchorage-independent proliferation potential of the glioma U87MG and U251 cell lines. (× 200) (**E**) The effect of HULC silencing on tube formation in the glioma U87MG and U251 cell lines. (× 200) (**F**) The effects of HULC silencing on the levels of VEGF-A, EGF, TGF-β1 and ESM-1 in the supernatants from the tube formation assays performed using the glioma U87MG and U251 cell lines. Error bars, ± SD; *P < 0.05. (**G**) The effects of HULC silencing on the protein expression of VEGFR-1/2, p-VEGFR-1/2, eNOS and EGFR in the glioma U87MG and U251 cell lines. (**H**) The effects of HULC silencing on the mRNA expression of VEGFR-1/2, eNOS and EGFR in the glioma U87MG and U251 cell lines.

To investigate the role of ESM-1 in the regulation of cancer malignancy by HULC, we transfected the ESM-1 expression plasmid into HULC-silenced U87MG and U251 cells to observe the reversed effect of HULC silencing in a subsequent experiment. We found that HULC silencing was suppress the expression of HULC in the U87MG and U251 cell lines; however, there was no significant change in HULC expression associated with the transfection of the ESM-1 expression plasmid (Figure 2A). We also observed decreased proliferation (including anchorage-independent proliferation) (Figure 2B–2D) and angiogenesis (Figure 2E). Additionally, the contents of VEGF-A, EGF, active TGF-β1 and ESM-1 (Figure 2F) and the expression of VEGFR-1/2, endothelial nitric oxide synthase (eNOS) and epidermal growth factor receptor (EGFR) (Figure 2G–2H) were significantly down-regulated in the HULC-silenced U87MG and U251 cell lines. However, ESM-1 expression could reverse the above-mentioned effects, suggesting that HULC promotes tumor proliferation and angiogenesis through ESM-1.

### HULC silencing suppresses the potential for the invasion, adhesion and migration of glioma cells *in vitro*

**Figure 3:**
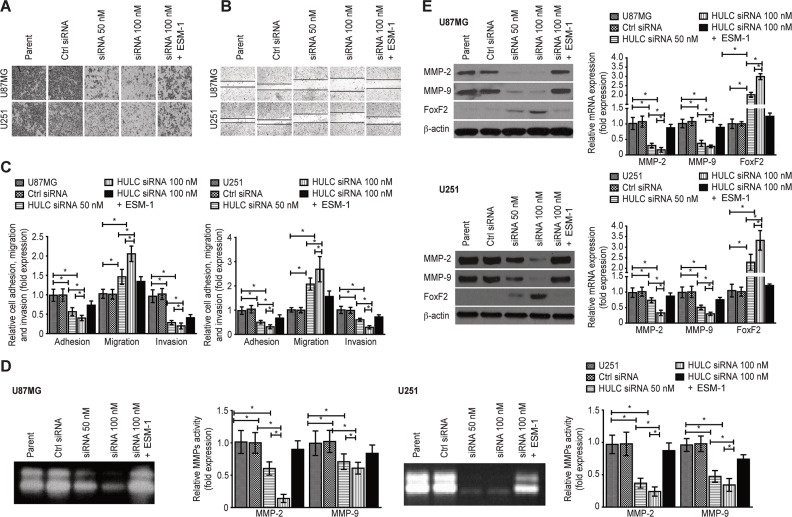
Effects of HULC on the invasion, adhesion and migration potential of glioma cells *in vitro* (**A**) The effect of HULC silencing on the invasion potential of the glioma U87MG and U251 cell lines. (**B**) The effect of HULC silencing on the migration potential of the glioma U87MG and U251 cell lines. (**C**) Graph showing the effects of HULC silencing on the adhesion, migration and invasion potential of the glioma U87MG and U251 cell lines. (**D**) The effect of HULC silencing on the expression of MMP-2/9 in the glioma U87MG and U251 cell lines. (**E**) The effect of HULC silencing on the activity of MMP-2/9 in the glioma U87MG and U251 cell lines. Error bars, ± SD; *P < 0.05.

We observed decreased invasion (Figure 3A), migration (Figure 3B) and adhesion (Figure 3C) in the HULC-silenced U87MG and U251 cell lines. There was decreased activity of MMP-2/9 in HULC-silenced U87MG and U251 cells (Figure 3D). Furthermore, the expression of MMP-2/9 was down-regulated and that of Forkhead box F2 (FoxF2) was up-regulated by HULC silencing in U87MG and U251 cells (Figure 3E). Again, above-mentioned effects were reversed by ESM-1 expression in HULC-silenced U87MG and U251 cells.

### HULC silencing blocks the cell cycle and induces anoikis in glioma cells *in vitro*

**Figure 4:**
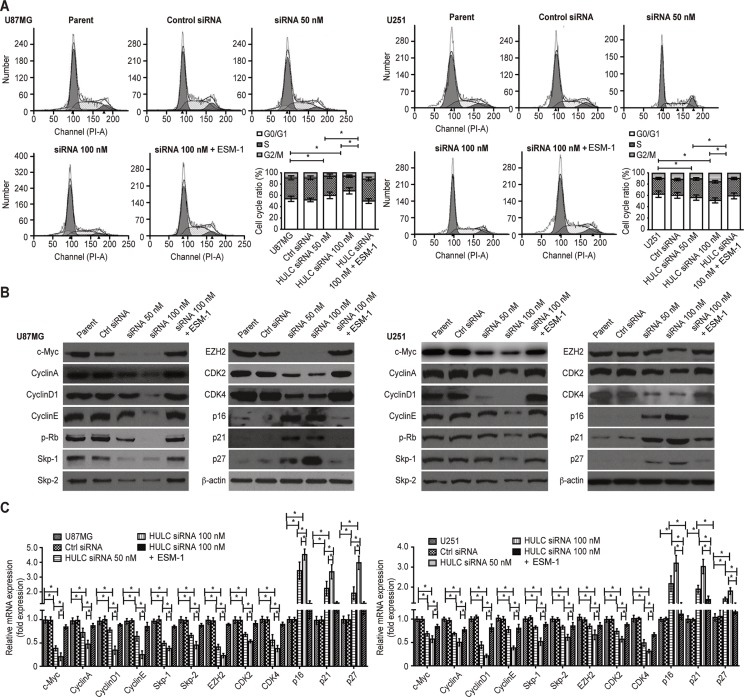
Effect of HULC on the cell cycle of glioma cells *in vitro* (**A**) HULC silencing arrested the cell cycle at G1/S phase in the glioma U87MG and U251 cell lines. (**B**) The effects of HULC silencing on the protein expression of c-Myc, Cyclin A/D1/E, p-Rb, Skp-1/2, CDK2/4, p16/21/27 and EZH2 in the glioma U87MG and U251 cell lines. (**C**) The effects of HULC silencing on the mRNA expression of c-Myc, Cyclin A/D1/E, p-Rb, Skp-1/2, CDK2/4, p16/21/27 and EZH2 in the glioma U87MG and U251 cell lines.

**Figure 5:**
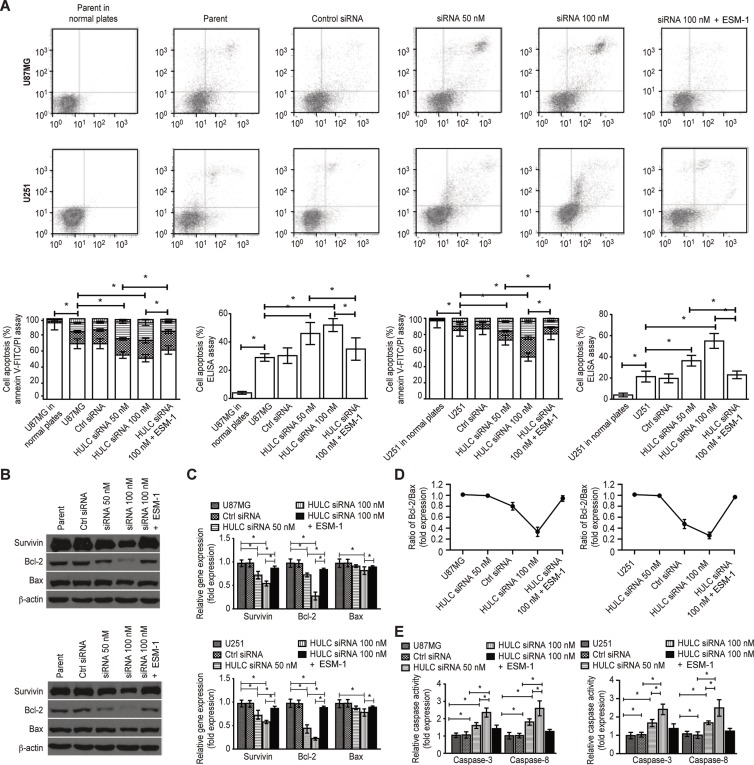
Effect of HULC on the anoikis of glioma cells *in vitro* (**A**) HULC silencing induced anoikis in the glioma U87MG and U251 cell lines. (**B**) The effects of HULC silencing on the protein expression of Survivin, Bcl-2 and Bax in the glioma U87MG and U251 cell lines. (**C**) The effects of HULC silencing on the mRNA expression of Survivin, Bcl-2 and Bax in the glioma U87MG and U251 cell lines. (**D**) The effect of HULC silencing on the ratio of Bcl-2/Bax in the glioma U87MG and U251 cell lines. (**E**) The effect of HULC silencing on the activity of caspase-3/8 in the glioma U87MG and U251 cell lines. Error bars, ± SD; *P < 0.05.

We found that the cell cycle was arrested in G1/S phase (Figure 4A) and that anoikis was induced (Figure 5A) in the HULC-silenced U87MG and U251 cell lines. The expression of Survivin, c-Myc, Cyclin A/D1/E, p-Rb, Skp-1/2, CDK2/4 and EZH2 (Figure 4B–4C and Figure 5B–5C) and the ratio of Bcl-2/Bax were significantly down-regulated (Figure 5D). However, the expression levels of p16, p21 and p27 (Figure 4B–4C) and the activity of caspase-3/8 were up-regulated by HULC silencing in U87MG and U251 cells (Figure 5E). ESM-1 expression again reversed the above-mentioned effects, suggesting that HULC promotes tumor malignancy through the cell cycle and anoikis through ESM-1.

### HULC up-regulates ESM-1 through the PI3K/Akt/mTOR signaling pathway *in vitro*

**Figure 6:**
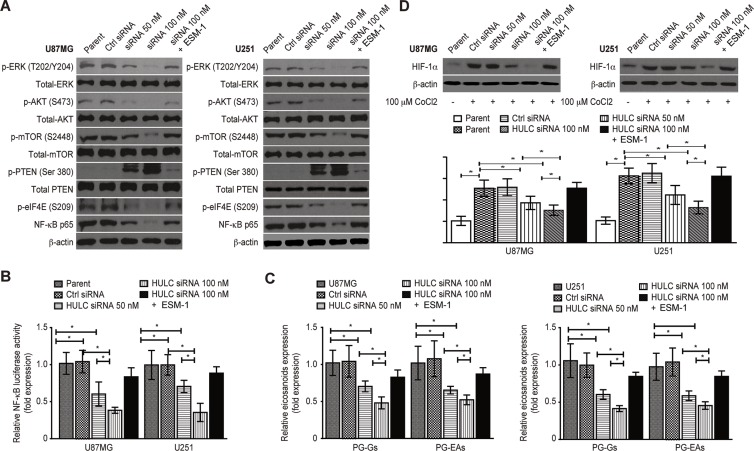
HULC activates the PI3K/Akt/mTOR signaling pathway and up-regulates the expression of HIF-1α in a hypoxic environment *in vitro* (**A**) The effect of HULC silencing on the phosphorylation of ERK, PTEN, AKT, mTOR and eIF4E and the expression of NF-κB p65 in the glioma U87MG and U251 cell lines. (**B**) The effect of HULC silencing onthe transcriptional activity of NF-κB p65 in the glioma U87MG and U251 cell lines. (**C**) The effect of HULC silencing on the contents of PG-Gs and PG-EAs in the glioma U87MG and U251 cell lines. (**D**) The effect of HULC silencing on the expression of HIF-1α in the glioma U87MG and U251 cell lines in a hypoxic environment induced by treatment with 100 μM CoCl_2_.

We found that HULC silencing decreased the phosphorylation of ERK, AKT, mTOR and the downstream molecule eukaryotic initiation factor 4E (eIF4E) (Figure 6A). The expression and transcriptional activity of NF-κB (p65) (Figure 6B) and the contents of prostaglandin glycerol esters (PG-Gs) and prostaglandin ethanolamides (PG-EAs) (Figure 6C), which are related to tumor malignancy, were improved by the phosphorylation of phosphatase and tensin homolog (PTEN) in the U87MG and U251 cell lines (Figure 6A).

A hypoxic environment is a major reason of tumor angiogenesis. Accordingly, we further investigated the role of HULC in angiogenesis in a hypoxic environment in glioma cells through treatment with 100 μM CoCl_2_ for 24 h to induce a hypoxic environment. We found that the expression of pro-angiogenesis genes was related to signaling molecules such as hypoxia inducing factor-1α (HIF-1α), which was improved. However, the expression of HIF-1α was reduced by HULC silencing, suggesting that HULC also activated the PI3K/Akt/mTOR pathway in the hypoxic environment (Figure 6D).

## DISCUSSION

Gliomas are a type of tumor that arises from glial cells and account for approximately 30% of all brain and central nervous system tumors and 80% of all malignant brain tumors [12]. Invasion is a major obstacle to therapy and management [13–14]. Many types of tumors, including gliomas, can develop vascularization via angiogenesis to provide an alternative blood supply pathway, which is also a key tumor aggravation process. As others long noncoding RNA (lncRNA), HULC has been shown to act as an oncogene in human hepatic carcinoma, esophageal cancer, osteosarcoma and pancreatic cancer [15]. However, its pro-invasion and pro-angiogenesis potential and its role in the PI3K/Akt/mTOR signaling pathway have not been previously explored.

Because higher expression of the HULC lncRNA is correlated with a higher glioma grade according to pathology, we suspected that HULC might promote the aggravation of gliomas. Furthermore, there was a positive correlation between the level of HULC, ESM-1, MVD (CD34) and VEGF in glioma patient tissue, suggesting that there was a potential relationship between HULC, ESM-1 and angiogenesis in the tumor progression.

To explore the mechanism underlying the regulation of glioma angiogenesis by HULC, we silenced HULC in U87MG and U251 cells and found that HULC silencing reversed the potential for glioma angiogenesis and influenced key processes such as proliferation (including anchorage-independent proliferation), migration, adhesion, invasion, the cell cycle (arrested in G1/S phase) and anoikis *in vitro*.

HULC induced tumor proliferation, migration and cell cycle progression by modulating the expression of Cyclin A/D1/E, p-Rb, c-Myc, CDK2/4, and p16/p21/27, which are targets of Skp-1/2 and inhibitors of CDK2/4 and EZH2. Adhesion and invasion were also promoted by the expression and activity of matrix metalloproteinases-2/9 (MMP-2/9) and the oncosuppressor FoxF2 [16–22], indicating that HULC also affect angiogenesis through cell cycle regulation and invasion.

Anoikis resistance is a critical mechanism in which cancer cells gain the ability to invade, which is key step in tumor metastasis [23]. The flow cytometry assay performed in the present study showed that anoikis resistance was suppressed in the HULC-silenced U87MG and U251 cell lines, suggesting that HULC was correlated with glioma anoikis resistance through the regulation of the Bcl-2/Bax ratio and caspase-3/8 activity.

VEGF-A and its receptor VEGFR-1/2 interact to affect the vascular permeabilization and mobilization of tumor cells by activating the production of eNOS. EGF/EGFR has been shown to present similar activity in other studies [24, 25]. We also found that HULC silencing suppressed VEGF/VEGFR and EGF/EGFR in the U87MG and U251 cell lines, suggesting a potential mechanism for the regulation of angiogenesis by HULC.

We further explored the effect of HULC on the PI3K/Akt/mTOR signaling pathway and found that HULC silencing decreased the content of TGF-β1; the phosphorylation of ERK, AKT, mTOR and the downstream molecule eukaryotic initiation factor 4E (eIF4E); and the transcriptional activity of NF-κB. However, the phosphorylation of PTEN was increased. Therefore, the PI3K/Akt/mTOR signaling pathway was the key component in the function of HULC in tumorigenesis via TGF-β1 activation. A hypoxic environment is a stimulus factor for tumor angiogenesis, and hypoxia inducing factor-1α (HIF-1α) is a key hypoxic response regulator that promotes the expression of VEGF-A, which is also a downstream component of the PI3K/Akt/mTOR signaling pathway [26–28]. We found that the expression of HIF-1α was reversed by HULC silencing, with higher expression being observed in a hypoxic environment, suggesting that HULC was also mediate angiogenesis by regulating the VEGF-A axis and the PI3K/Akt/mTOR signaling pathway in a hypoxic environment. Moreover, it has been demonstrated that eicosanoids, PG-Gs and PG-EAs, are biologically active lipids that serve as HULC effectors. Therefore, we considered there was a relationship between the expression of eicosanoids and HULC in gliomas.

Importantly, we first discovered that HULC and ESM-1, which are up-regulated in multiple types of aggressive human cancers and create a bridge between VEGF and VEGFR to induce biological activity and contribute to cancer malignancy, including angiogenesis and invasion [29, 30], are frequently overexpressed in glioma patient tissues and positively correlated with the malignancy of patients. Additionally, we found that HULC silencing could suppress the expression of ESM-1 in the glioma U87MG and U251 cell lines. However, the effects of HULC silencing was reversed by ESM-1 expression. However, there was also some question, for example, (1) whether VEGF generate the same results as ESM-1 on HULC silenced cells; (2) whether the effects of ESM-1 be blocked by its antibody such as LFA1; (3) whether there was another effect of hypoxic mimicry with cobalt chloride on glioma biological behavior and what's the effect of HULC silencing on it, which need to further explored.

## MATERIALS AND METHODS

### Patient specimens and cell lines

A total ofssss 120 patients with glioma, 30 of whom were staged as WHO grades II-IV, were enrolled in this study (Tianjin Huanhu Hospital, Tianjin, China), and their patient information is presented in Supplementary Table S1. The mean age of the patients was 45.7 years (range, 13–72). Glioma specimens were obtained from patients treated at the Department of Neurosurgery of Tianjin Huanhu Hospital in China. The study was approved by the Institute Research Ethics Committee of Tianjin Huanhu Hospital. The clinical data for the glioma patients are listed in Table 2.

**Table 2:** Glioma patient clinical data

Variable		Tumor WHO grade		
Grade		II	III	IV
Age	≥ 40 years	20	26^*^	34^*, **^
	< 40 years	20	14^*^	6^*, **^
Gender	Male	22	25	28
	Female	18	15	12

* compared with grade II, *P* < 0.05;

** compared with grade III, *P* < 0.05.

The human glioma U87MG and U251 cell lines were obtained from the American Type Culture Collection (USA) and maintained at 37°C in a humidified atmosphere with 5% CO_2_, with 100 μM cobalt chloride (CoCl_2_) being added to induce a hypoxic environment [11]. The culture medium was DMEM medium with 10% fetal bovine serum (FBS) (Gibco). All cells were stored in liquid nitrogen and used for experiments within 6 months.

Cells were cultured in a 6-well plate for 24 h and transfected with siRNAs or expression plasmids using GeneJuice (Merck) according to the manufacturer's protocol for another 6 h. Then the fresh culture medium was added and cells were harvested after 72 h in all experiments. The HULC siRNA (3′-CCUCCAGAACUGUGAUCCAdTdT-5′) for the HULC siRNA group (50 nM and 100 nM), scrambled siRNA for the control siRNA (Ctrl siRNA) group and NF-κB p65 CRISPR Activation Plasmid was purchased from Santa Curz (USA), ESM-1 expression plasmid (pcDNA3.1-ESM-1) was constructed by our laboratory.

### Immunohistochemical analysis

Consecutive sections of formalin-fixed, paraffin-embedded (FFPE) tumor samples were subjected to immunohistochemical (IHC) analysis for VEGF-A, microvessel density (MVD) (CD34) and ESM-1 using a 3, 3′-diaminobenzidine (DAB) substrate kit (Maxin, Fuzhou, China) according to the manufacturer's instructions; 500 cells (× 400) were counted. The results were scored as previously described by two pathologists, who were blinded to the clinicopathology data, and analyzed according to the label index. Label index (%) *=* count (positive cell)/count (total cell) × 100%.

### Cell proliferation assay

A 0.1 ml aliquot of a cell suspension (3 × 10^4^/ml) was seeded into each well of a 96-well microplate, followed by incubation for 72 h under standard conditions. The BrdU cell proliferation kit (Abcam) was used to determine cell numbers every 24 h according to the manufacturer's instructions using a microplate reader (Thermo Scientific Multiskan FC). Morphological changes were photographed using a microscope after 72 h.

### Cell anchorage-independent assay

The anchorage-independent growth assay was measured using a methylcellulose cell clone kit (Haling, Shanghai, China) according to the manufacturer's instructions. Briefly, 1 × 10^4^ cells were harvested and resuspended in top agar for 24 h at 37°C in a humidified atmosphere with 5% CO_2_. Then the colonies were counted after 7 days and medium was refreshed 2 times a week.

### Tube formation assay

Matrigel (BD Biosciences) was prepared at 4°C and used to coat a 24-well plate. The gel was allowed to solidify for 60 min at 37°C, and then 5 × 10^4^ cells were added to the medium in each well, followed by incubation at 37°C for 6 h. Tube formation was observed with a microscope (× 200).

### Liquidchip assay

The levels of VEGF-A, epidermal growth factor (EGF), active transforming growth factor-β1 (TGF-β1) and ESM-1 in the supernatants from the tube formation assay were measured using a liquidchip assay according to the manufacturer's instructions (R & D Systems). The concentration of each cytokine was determined using a standard curve according to the kit's instructions.

### Migration, invasion, adhesion and cell cycle analysis *in vitro*

A migration assay was performed via the scratch method. A total of 3 × 10^5^ cells per well were incubated in a 6-well-microplate overnight, and a single scarification was performed by 50 μl pipet. Then, the cells were washed and media were refreshed, and the cells were incubated for another 72 h under standard conditions. Scarification healing was analyzed using a microscope.

Cell invasion was assessed by cell invasion assay kit (Cell Biolabs) according to the manufacturer's instructions. Briefly, 3 × 10^6^ cells were suspended in serum-free medium and added into the insert. At same time, 500 μl of medium with 10% BSA was added to the lower well of the plate. The non-migratory cells were removed and the migratory cells were stained by crystal violet and counted by microscope after incubated for 72 h.

To investigate cell adhesion on recombinant collagen, a 96-well microplate was coated via incubation with 100 μl per well of a recombinant collagen solution (50 μg/ml) in serum-free DMEM for 1 h. A total of 1 × 10^5^ cells per well were incubated for 1 h under standard conditions. The medium and non-adherent cells were then aspirated, and the wells were washed three times with phosphate-buffered saline (PBS). Absorption was measured via the MTS assay at 490 nm using a microplate reader (Thermo Scientific Multiskan FC).

Cell cycle analysis was performed using a flow cytometry assay. Cells were harvested and fixed with 70% cold ethanol at 4°C overnight. Then, the cells were washed with PBS and stained with propidium iodide (PI) with RNaseA. The cell cycle distribution was assessed using a FACSAria flow cytometer (BD Biosciences) at 488 nm.

### Anoikis assay

A total of 5 × 10^3^ cells were incubated in 6-well cell low attachment plates (Corning) for 72 h under standard conditions. Cells were then harvested, and anoikis was assessed using an Annexin V-FITC/PI cell apoptosis kit (BD Biosciences), a cell death ELISA (DNA fragmentation, Roche), and a FACSAria flow cytometer (BD Biosciences), all according to the manufacturers' instructions. The group of cells cultured in normal plates was considered the control group.

### Caspase-3/8 activity assay

To investigate the cellular activity of caspase-3/8, 5 × 10^3^ cells were incubated in 6-well cell low attachment plates for 72 h under standard conditions, and the cellular activity of caspase-3/8 was measured according to the manual's instruction (FLICA Caspase Detection Kits, ImmunoChemistry Technologies) using a microplate reader.

### Western blotting assay

Total proteins were obtained through RIPA lysis buffer (Millipore) extraction and centrifugation at 12,000 g for 10 min. The total protein concentrations were measured with a bicinchoninic acid (BCA) kit (Sigma Aldrich). A 100 μg sample of the proteins was separated via 12% SDS-PAGE and transferred onto a polyvinylidene fluoride (PVDF) membrane and blocked by 1% BSA. Target proteins were detected through incubation overnight at 4°C with primary antibodies (Supplementary Table S2). The membranes were then washed and incubated for 1 h with peroxidase-labeled anti-rabbit IgG (Santa Cruz, diluted at 1:2000). Then, the membranes were washed 3 times with PBST (Phosphate Buffer Solution with 0.05% Tween20) and exposed to the Immobilon^™^ Western chemiluminescent horseradish peroxidase (HRP) substrate (Millipore) for visualization. β-actin was used as the control.

### Real-time PCR assay

FFPE tissue samples was cut by 5–10 μm and 1ml xylene was added, quaked 10s, centrifugated at 12000 rpm × 2 min, discarded supernatant, and 1ml alcohol was added, centrifugated at 12000 rpm × 2 min, discarded supernatant, then 10 μL Proteinase K was added and total RNA in paraffin-embedded (FFPE) tissue samples was extracted using the RNApure FFPE Kit (Cwbiotech, Beijing, China) according to the manual's instructions.

Cell total RNA was extracted from the cultured cells using the TRIzol reagent (Invitrogen). Total RNA from paraffin blocks was extracted using the Recover AII^™^ Total Nucleic Acid Isolation kit (Invitrogen) according to the manual's instructions. Total RNA was then reverse transcribed and mRNA expression of the target genes were detected using real-time PCR assay. The PCR conditions were as follows: denaturation at 95°C for 10 min, followed by 40 cycles at 95°C for 15 s, 60°C for 60 s and a final elongation at 95°C for 15 s, 60°C for 60 s and 95°C for 15 s. The expression levels of the genes were normalized to that of the housekeeping gene β-actin, as a control. The full details of the primers used in these experiments are shown in Supplementary Table S3.

### Zymography assay

The activity of MMP-2/9 in the supernatants from the 3D cultures was measured using a zymography assay. Supernatants were collected, concentrated via ultrafiltration (Amicon), and subjected to 10% SDS-PAGE (including 0.01% (wt/vol) gelatin as a substrate). The gels were agitated in 2.5% Triton X-100 for 60 min and incubated in zymogram developing buffer (50 mM Tris-HCl (pH 7.5), 10 mM CaCl_2_, 150 mM NaCl, 1 mM ZnCl_2_, and 0.02% NaN_3_) for 40 hours at 37°C. The gels were subsequently stained with Coomassie Blue R-250 for 30 min and destained until matrix metalloproteinase (MMP) activity appeared as clear bands against a dark blue background.

### Extraction and quantization of eicosanoids

A total of 3 × 10^5^ cells per well were incubated in a 6-well microplate for 72 h under standard conditions. Then, the cells were starved in serum-free medium for 4 h to minimize the contribution of serum-derived metabolites, washed twice with PBS and harvested via scraping and centrifugation. The cell pellets were flash frozen at −80°C, and glycerol esters (PG-Gs) and prostaglandin ethanolamides (PG-EAs) were extracted and analyzed using liquid chromatography/tandem mass spectrometry (LC-MS/MS).

### Reporter gene assay

A mixture of an inducible ESM-1 luciferase plasmid and a constitutively expressed *Renilla* luciferase plasmid, as the control, was purchased from Qiagen Co. Ltd. A total of 3 × 10^5^ cells per well were incubated in a 6-well microplate overnight, and the above-mentioned plasmids were transfected for 6 h using GeneJuice (Merck) according to the manufacturer's protocol. Then, the media were refreshed, and the cells were incubated for another 72 h under standard conditions. The resultant luciferase activity was analyzed using the Bright-Glo^™^ Luciferase Assay System (Promega).

### Statistical analysis

SPSS version 13.0 (SPSS Inc., IL, USA) was used for statistical analysis. The statistical analysis was performed using ANOVA with the Tukey-Kramer multiple comparisons test. A *p* value < 0.05 was considered statistically significant. All calculations were performed using GraphPad Prism 6.0 software (GraphPad Software Inc.).

## SUPPLEMENTARY MATERIAL TABLES


