# Supplementary material for: HULC long noncoding RNA silencing suppresses angiogenesis by regulating ESM-1 via the PI3K/Akt/mTOR signaling pathway in human gliomas
Source: Oncotarget. 2016 Feb 15;7(12):14429–40. doi: 10.18632/oncotarget.7418 (PMC4924726; doi:10.18632/oncotarget.7418)
Supplement: Supplementary file 1 [file oncotarget-07-14429-s001.pdf]

HULC long noncoding RNA silencing suppresses angiogenesis by regulating ESM-1 via the PI3K/Akt/mTOR signaling pathway in human gliomas

Supplementary Materials

Supplementary Table S1

Grade II	No	gender	age	ESM-1	CD34	VEGF	HULC/β-actin
	1	female	53	21	29.80	39	2.1295
	2	female	43	25	22.70	44	2.9182
	3	male	38	38	34.30	46	3.1548
	4	male	44	39	14.90	10	3.2336
	5	male	28	10	39.30	17	1.1830
	6	male	51	48	13.60	14	2.6027
	7	female	43	44	19.20	26	1.5774
	8	male	43	40	36.00	31	3.0759
	9	male	26	42	35.10	33	2.3661
	10	male	38	40	28.60	45	0.6310
	11	male	51	19	16.90	19	2.0506
	12	male	41	18	34.20	43	2.7604
	13	male	23	11	17.50	40	1.7351
	14	male	49	9	44.60	36	3.2336
	15	female	53	36	19.60	26	3.3914
	16	female	27	7	20.20	8	2.2083
	17	female	35	28	38.00	12	2.6027
	18	female	33	31	38.50	9	1.4985
	19	female	56	39	26.00	12	2.4449
	20	male	19	55	17.60	37	3.1548
	21	female	47	27	21.30	25	1.6136
	22	male	28	37	36.90	36	2.2112
	23	female	51	40	34.10	23	2.3905
	24	female	30	41	29.60	39	2.4502
	25	male	39	15	17.30	23	0.8964
	26	male	41	33	36.20	45	1.9721
	27	male	30	20	19.50	30	1.4079
	28	female	38	39	28.50	16	3.0446
	29	female	50	30	37.40	14	2.3420

	30	male	33	8	17.60	23	0.6245
	31	male	36	26	46.20	25	2.0297
	32	male	39	35	16.10	29	2.7323
	33	female	42	22	15.90	22	1.7175
	34	male	39	41	35.70	14	2.6930
	35	female	49	43	31.40	38	2.8244
	36	male	46	28	17.30	31	1.4172
	37	female	43	33	24.50	44	1.6703
	38	female	30	19	29.30	28	0.9617
	39	male	47	31	39.00	13	1.8412
	40	female	35	40	37.50	11	2.3758

Grade III	No	gender	age	ESM-1	CD34	VEGF	HULC/β-actin
	1	male	69	20	37.50	57	5.3704
	2	male	17	22	44.80	53	8.5032
	3	male	32	21	30.60	54	12.9785
	4	male	45	33	36.80	39	14.5449
	5	male	43	11	54.50	33	11.1884
	6	male	25	35	58.10	22	12.3072
	7	male	43	36	38.30	19	2.9090
	8	male	60	34	25.70	43	2.6852
	9	female	59	19	30.00	45	14.0974
	10	male	50	46	48.40	41	13.6498
	11	male	35	48	38.00	43	13.2023
	12	female	34	49	21.00	38	12.5310
	13	male	57	29	53.00	48	12.9785
	14	female	13	42	44.00	50	13.6498
	15	male	55	41	34.00	56	10.5171
	16	male	41	39	29.00	62	13.2023
	17	male	65	40	54.00	54	5.1467
	18	female	66	48	52.60	37	16.1113
	19	female	32	23	47.00	36	12.5310
	20	female	45	36	23.20	28	7.1606
	21	male	16	40	35.00	48	7.9050
	22	male	48	51	56.00	34	11.0659
	23	male	30	36	47.00	55	7.8112

	24	male	56	41	36.00	41	8.8961
	25	female	40	37	27.00	46	6.7653
	26	female	29	10	51.00	43	1.8285
	27	female	56	24	56.00	57	8.7767
	28	female	36	38	48.00	42	4.3883
	29	female	26	58	32.00	36	5.7665
	30	male	24	65	35.00	25	8.8014
	31	male	43	50	31.00	42	9.8637
	32	female	40	55	44.40	30	7.5874
	33	male	48	13	34.90	58	14.5281
	34	male	41	12	19.20	46	3.4339
	35	female	39	63	52.30	31	3.1698
	36	female	44	61	39.40	23	11.5164
	37	female	66	59	42.60	29	11.1508
	38	male	61	56	41.50	21	10.7852
	39	male	56	58	30.00	35	15.2863
	40	male	50	61	44.00	54	10.9609

Grade IV	No	gender	age	ESM-1	CD34	VEGF	HULC/β-actin
	1	female	63	60	36.10	35	20.4105
	2	male	63	44	58.00	53	10.8237
	3	male	72	43	49.00	44	8.3497
	4	male	60	45	50.00	47	10.2052
	5	male	59	44	62.40	42	16.6995
	6	male	56	23	44.60	57	18.5550
	7	male	57	56	54.70	42	12.9885
	8	male	58	51	66.00	66	13.6070
	9	female	51	41	47.00	59	20.1012
	10	male	42	33	41.20	57	20.4105
	11	male	44	45	57.30	62	11.1330
	12	male	68	60	42.00	65	7.7312
	13	male	60	60	66.50	49	20.4105
	14	male	39	51	60.20	52	14.8440
	15	male	67	42	40.60	38	13.9162
	16	male	32	43	59.20	43	18.8642
	17	female	61	63	63.80	64	17.6272
	18	female	28	29	63.00	54	11.7515
	19	female	47	74	55.70	63	8.9682
	20	female	58	40	44.00	35	11.7515
	21	male	62	66	65.00	63	17.3541
	22	male	55	35	46.00	46	8.2358
	23	female	67	27	64.00	65	5.8827
	24	male	51	33	57.00	36	7.6476
	25	male	35	54	69.00	59	13.8244
	26	female	59	60	43.00	60	15.5892
	27	male	49	42	33.00	47	2.1350
	28	female	29	44	65.30	55	11.1837
	29	male	54	65	37.00	53	17.5312
	30	female	61	66	60.90	50	18.4380
	31	male	59	36	34.00	48	9.3701
	32	male	52	25	40.00	49	6.0452
	33	male	60	66	49.00	52	18.4380
	34	female	31	48	51.00	64	12.9973
	35	male	60	45	56.00	51	11.4860
	36	male	55	61	56.00	48	17.9154
	37	female	61	57	47.00	35	12.8226
	38	male	56	38	66.00	66	9.2323
	39	male	63	29	60.00	64	6.9242
	40	male	57	38	44.00	57	9.2323

Supplementary Table S2: The anti-body used in the study

No.	gene	Manufacturer	Cat #	Ratio
1	ESM-1	Santa Cruz Biotechnology	sc-20344	1:1500
2	ESM-1 (IHC)	Santa Cruz Biotechnology	sc-20344	1:100
3	CD34 (IHC)	Santa Cruz Biotechnology	sc-133082	1:100
4	VEGF (IHC)	Santa Cruz Biotechnology	sc-152	1:100
5	MMP-2	Santa Cruz Biotechnology	sc-13594	1:1500
6	MMP-9	Santa Cruz Biotechnology	sc-21733	1:1500
7	Survivin	Santa Cruz Biotechnology	SC-8806	1:1500
8	Bcl-2	Santa Cruz Biotechnology	SC-7382	1:1500
	Bax	Santa Cruz Biotechnology	Sc-493	1:1500
9	c-Myc	Santa Cruz Biotechnology	sc-40	1:1500
10	CDK2	Santa Cruz Biotechnology	sc-163	1:1500
11	CDK4	Santa Cruz Biotechnology	sc-23896	1:1500
12	CyclinA	Cell Signaling Technology	4656	1:1500
13	CyclinD1	Cell Signaling Technology	2926	1:1500
14	CyclinE	Cell Signaling Technology	2925	1:1500
15	EZH2	Santa Cruz Biotechnology	sc-25383	1:1500
16	Skp1	Cell Signaling Technology	2156	1:1500
17	Skp2	Cell Signaling Technology	2652	1:1500
18	p16	Santa Cruz Biotechnology	sc-377412	1:1500
19	p21	Cell Signaling Technology	2947	1:1500
20	p27	Cell Signaling Technology	3686	1:1500
21	p-Rb	Santa Cruz Biotechnology	9307	1:1000
22	HIF-1α	Santa Cruz Biotechnology	sc-13515	1:1500
23	FoxF2	Santa Cruz Biotechnology	sc-133590	1:1500
24	eNOS	Santa Cruz Biotechnology	sc-137111	1:1500
25	EGFR	Santa Cruz Biotechnology	sc-373746	1:1500
26	VEGFR-1	Cell Signaling Technology	2893	1:1500
27	VEGFR-2	Cell Signaling Technology	2479	1:1500
28	p-VEGFR-1	Santa Cruz Biotechnology	sc-101819	1:1000
29	p-VEGFR-2	Cell Signaling Technology	2478	1:1000
30	mTOR	Cell Signaling Technology	2983	1:1500
31	p-mTOR	Cell Signaling Technology	5536	1:1000
32	p-eIF4E	Cell Signaling Technology	9741	1:1000
33	Akt	Cell Signaling Technology	9272	1:1500
34	p-Akt	Cell Signaling Technology	4060	1:1000
35	p65	Santa Cruz Biotechnology	sc-372	1:1500
36	p-PTEN	Santa Cruz Biotechnology	sc-377573	1:1000
37	p-ERK	Cell Signaling Technology	4370	1:1000

38	β-Actin	Sigma Aldrich	A228	1:5000
39	Anti-rabbit IgG, HRP-linked Antibody	Cell Signaling Technology	7074	1:2000
40	Anti-mouse IgG, HRP-linked Antibody	Cell Signaling Technology	7076	1:2000
41	Anti-Goat IgG, HRP-linked Antibody	Sigma Aldrich	A5420	1:2000

Supplementary Table S3: The primers used in the study

Gene	Primer Sequences (5′-3′)
HULC	Forward: CAGGAAGAGTCGTCACGAGAACCAG
	Reverse:CTTCTTGCTTGATGCTTTGGTCTGT
ESM-1	Forward: CAGGCATGGATGGCATGAAG
	Reverse: CTGACTGGCAGTTGCAGGTCTC
MMP-2	Forward: TGGATGGAGGAAAACCAAGCC
	Reverse: AGGGAGCAGAGATTCGGACTT
MMP-9	Forward: ACCACCACAACATCACCTA
	Reverse: ACCACAACTCGTCATCGT
Survivin	Forward: AGAACTGGCCCTTCTTGGAGG
	Reverse: CTTTTTATGTTCCTCTATGGGGTC
Bcl-2	Forward: ATGTGTGTGGAGAGCGTCAA
	Reverse: ACAGTTCCACAAAGGCATCC
Bax	Forward: GGGGACGAACTGGACAGTAA
	Reverse: CAGTTGAAGTTGCCGTCAGA
c-Myc	Forward: GAAATTGGGAACTCCGTGTG
	Reverse: CTAGGGCGAGAGGGAGGTT
HIF-1α	Forward: TCACCACAGGACAGTACAGGATGC
	Reverse: CCAGCAAAGTTAAAGCATCAGGTTCC
CDK2	Forward: GCCAGAAACAAGTTGACGGGAGA
	Reverse: TGGGTGTAAGTACGAACAGGGAC
CDK4	Forward: CTGTGGACATGTGGAGTGTTG
	Reverse: GGCAGAGATTCGCTTGTGTG
CyclinA	Forward: GCCATTAGTTTACCTGGACCCAGA
	Reverse: CACTGACATGGAAGACAGGAACCT
CyclinD1	Forward: TGAACTACCTGGACCGCT
	Reverse: GCCTCTGGCATTTTGGAG
CyclinE	Forward: TTCCACACAGGAGCAAAGTATG
	Reverse: TGCAACTTTGGAGGGTAGATTT

EZH2	Forward: GCCAGACTGGGAAGAAATCTG
	Reverse: TGTGCTGGAAAATCCAAGTCA
Skp1	Forward: CTCCCGAGGAAATACGCAAG
	Reverse: CGGGCGAAAAGTCCTTCTTA
Skp2	Forward: GCTGCTAAAGGTCTCTGGTGT
	Reverse: AGGCTTAGATTCTGCAACTTG
p16	Forward: AGGGGTTGGTTGGTTATTAG
	Reverse: CTACCTACTCTCCCCCTCTC
p21	Forward: GCGACTGTGATGCGCTAAT
	Reverse: TAGGGTTCCTCTTGGAGAA
p27	Forward: TGCAACCGACGATTCTTCTACTCAA
	Reverse: CAAGCAGTGATGTATCTGATAAACAAGGA
p65	Forward: GCGAGAGGAGCACAGATACC
	Reverse: GATGCGCTGAGTGATAGCCT
FoxF2	Forward: CACTACTGGACCATCGACCC
	Reverse: CTCACCACGCGGTGGTACAT
TGF-β1	Forward: GACTACTACGCCAAGGAG
	Reverse: TGAGGTATCGCCAGGAAT
eNOS	Forward: GCACAGGAAATGTTCACCTAC
	Reverse: CACGATGGTGACTTTGGCTAG
EGF	Forward: AGCCAGCTCTGATCTAATCTGG
	Reverse: TTTTGCAAATATGTTCACAGCC
EGFR	Forward: GTCTCTTGCCGGAATGTCAG
	Reverse: CTCACCCTCCAGAAGGTTGC
VEGF-A	Forward: CTACCTCCACCATGCCAAGT
	Reverse: GCAGTAGCTGCGCTGATAGA
VEGFR-1	Forward: CGGAAGGAAGACAGCTCATC
	Reverse: CTTCACGCGACAGGTGTAGA
VEGFR-2	Forward: GCTTTCGGTAGTGGGATGAA
	Reverse: GGAATCCATAGGCGAGATCA
β-actin	Forward: AGGCACCAGGGCGTGAT
	Reverse: GCCCACATAGGAATCCTTCTGAC
